# The costs of delivering emergency care at regional referral hospitals in Uganda: a micro-costing study

**DOI:** 10.1186/s12913-021-06197-7

**Published:** 2021-03-16

**Authors:** Kalin Werner, Tracy Kuo Lin, Nicholas Risko, Martha Osiro, Joseph Kalanzi, Lee Wallis

**Affiliations:** 1 Division of Emergency Medicine, University of Cape, Cape Town, South Africa; 2 Department of Social and Behavioral Sciences, Institute for Health & Aging, University of California, San Francisco, San Francisco, CA USA; 3 Johns Hopkins University School of Medicine, Baltimore, MD USA; 4 Makerere University School of Medicine, Kampala, Uganda

**Keywords:** Micro-costing, Emergency care, Bottom-up costing, LMIC

## Abstract

**Background:**

Uganda experiences a high morbidity and mortality burden due to conditions amenable to emergency care, yet few public hospitals have dedicated emergency units. As a result, little is known about the costs and effects of delivering lifesaving emergency care, hindering health systems planning, budgeting and prioritization exercises. To determine healthcare costs of emergency care services at public facilities in Uganda, we estimate the median cost of care for five sentinel conditions and 13 interventions.

**Methods:**

A direct, activity-based costing was carried out at five regional referral hospitals over a four-week period from September to October 2019. Hospital costs were determined using bottom-up micro-costing methodology from a provider perspective. Resource use was enumerated via observation and unit costs were derived from National Medical Stores lists. Cost per condition per patient and measures of central tendency for conditions and interventions were calculated. Kruskal-Wallis H-tests and Nemyeni post-hoc tests were conducted to determine significant differences between costs of the conditions.

**Results:**

Eight hundred seventy-two patient cases were captured with an overall median cost of care of $15.53 USD ($14.44 to $19.22). The median cost per condition was highest for post-partum haemorrhage at $17.25 ($15.02 to $21.36), followed by road traffic injuries at $15.96 ($14.51 to $20.30), asthma at $15.90 ($14.76 to $19.30), pneumonia at $15.55 ($14.65 to $20.12), and paediatric diarrhoea at $14.61 ($13.74 to $15.57). The median cost per intervention was highest for fracture reduction and splinting at $27.77 ($22.00 to $31.50). Cost values differ between sentinel conditions (*p* < 0.05) with treatments for paediatric diarrhoea having the lowest median cost of all conditions (*p* < 0.05).

**Conclusion:**

This study is the first to describe the direct costs of emergency care in hospitals in Uganda by observing the delivery of clinical services, using robust activity-based costing and time motion methodology. We find that emergency care interventions for key drivers of morbidity and mortality can be delivered at considerably lower costs than many priority health interventions. Further research assessing acute care delivery would be useful in planning wider health care delivery systems development.

**Supplementary Information:**

The online version contains supplementary material available at 10.1186/s12913-021-06197-7.

## Background

Emergency care (EC) encompasses a range of interventions delivered soon after the onset of acute disease or injury. Due to the time-sensitive nature of successfully managing these conditions, there is strong evidence that organized EC systems effectively reduce mortality and improve patient outcomes [1–4]. Globally, emergency conditions contribute to over half of total deaths, the majority of which occur in low- and middle-income countries (LMICs) where formal emergency care systems are rare [5–7]. Emergencies occur on a daily basis regardless of whether there is an organized effort to treat them, indicating that the consequences of neglecting EC systems may be disastrous [7, 8]. With 1191 deaths from emergency conditions per 100,000 and 52,441 DALYs from emergency conditions per 100,000 [7] the burden of emergency conditions in Uganda is devastating.

There is a mounting body of evidence demonstrating the impact of emergency care in LMICs [9, 10]; however, not enough is known about the cost and resources required to achieve these gains. The existing body of cost-effectiveness literature is methodologically weak, often failing to use empirically derived local inputs, and focused on analysing single-intervention rather than the cost-effectiveness of system changes and process improvements [11]. Electronic medical records, digital billing systems, and dedicated emergency unit budgets available in high-income settings have supported efforts to evaluate the costs for many EC interventions in these settings [12, 13]. However, data from high-income settings do not accurately reflect the fiscal environment more common in LMICs. Numerous additional research challenges, including: paper-based data management systems, unreliable supply chains with frequent stockouts and highly variable accounting practices, present further challenges in assessing local costs in LMIC environments to provide context-relevant guidance [14]. Under these circumstances, capturing accurate figures requires resource and time intensive methods such as direct observation via time-motion methodology – a likely reason for the paucity of published data surrounding performance and capability of delivering of EC in LMICs [15].

Nevertheless, it is problematic to pledge resources to strengthen EC delivery systems without first understanding the cost. For this reason, knowing the true outlay of EC is essential to understanding the affordability or sustainability of scaling up services given already strained health care budgets in many LMICs. Hospital managers and decision makers require this information to engage in evidence-based decision making while setting priorities and improving efficiencies. While efforts have been made to quantify the pattern of resource use and procedure utilization in functional emergency units in Uganda, the actual cost of delivering these services in this setting remains unclear [16].

In this study, we describe methods for performing an accurate costing of the provision of EC from a health care provider perspective at the facility level, illustrated through costing research performed at public facilities in Uganda. This study addresses some of the critical gaps in the economic evidence for EC in LMICs and accompanies ongoing research assessing the effectiveness of the World Health Organization (WHO) Emergency Care Toolkit in Uganda, whose methods are previously described [17]. To offer insights into the cost of implementing EC in an LMIC, a bottom-up micro-costing methodology is used to classify all relevant cost components of EC to the most exhaustive level by gathering detailed information on the quantity of resources used and their value at a patient-specific level. Precise cost estimates, which reflect the realities of resource use as it occurs in facility-based delivery of EC in a low-resource setting, are presented. These estimates may be used to demonstrate cost-effectiveness of EC interventions in future evidence-based decision making in resource scarce contexts.

## Methods

A direct, activity-based costing was carried out over a four-week period from September 2019 to October 2019. The cost analysis was conducted from the healthcare provider perspective following published methods guidelines on costing approaches performed in LMICs [18, 19]. All costs were collected in 2017 Ugandan shillings, and later adjusted and reported in 2017 US Dollars using the annual average exchange rate (1 dollar = 3700 shillings) [20]. Ethics approvals were obtained from the institutional review board at University of Cape Town and University of Makerere, and site approval was provided by the Uganda Ministry of Health (MoH) (Reference: 549/2019 & 2019–013).

### Setting

Data were collected by convenience sampling at five regional referral hospital sites (RRHs), which are tertiary level centres that offer both general and specialist clinical services and partake in teaching and research. The following criteria were used for site inclusion to the study: a public RRH, with an emergency/casualty/A&E unit, which had not received any of the elements of the WHO EC Toolkit. Any sites which had received previous elements of the WHO toolkit were excluded from the study. All sites are funded by the Ugandan government and deliver most services free of user charges. The five sites are geographically diverse and serve various catchment sizes. In particular, Mbale RRH and Jinja RRH receive high numbers of mass casualties from road traffic accidents due to proximity of the nearby highway. There are currently no national guidelines for the layout of emergency units [14]. Consequently, each site demonstrated unique features to their physical space, staffing, equipment and supplies. A brief overview of key characteristics of each of the five sites, observed during site visits is provided in the Table 1.

**Table 1:** Key characteristics of study sites

	Jinja	Mbale	Gulu	Hoima	Kabale
**Distance from Kampala (km)**	80 km E	224 km E	333 km N	200 km W	426 km SW
**Population served**	3.5 million	2 million	2 million	Over 3 million	2 million
**Region**	Eastern central region	Mid-eastern region	Mid-northern region	Mid-western region	Kigezi region
**Districts served**	Bugiri, Buyende, Iganga, Jinja, Kaliro, Kamuli, Kayunga, Luuka, Mayuge, Namutumba, Namayingo	Busia, Budaka, Bududa Kibuku, Kapchorwa, Kween Bukwo, Butalega, Manafwa, Mbale, Pallisa Sironko, Tororo	Amuru, Gulu, Kitgum, Lamwo, Pader, Nwyoa, Oyam	Hoima, Kibale, Masindi, Bulisa, Kiryandongo, Kyankwanzi, Kiboga, and Easter part of DRC	Kabale, Kisoro, Rukungiri, Kanungu and some parts of Ntungamo (as well as ppl from neighboring Rwanda and DRC)
**Estimated annual number of visits to EU**^a^	10,788 Inpatient	9156 Inpatient 9540 Outpatient	4452 Inpatient 8172 Outpatient	2952 Inpatient	2076 Inpatient 3240 Outpatient
**No. of beds in hospital**^b^	500	302	335	300	280
**No. of staff in EU department over 24 h period**^c^	8 Nurses 2 Doctors	7 Nurses 3 Interns 4 Clinical Officers 1 Medical Officer	3 Nurses 2 interns 1 Medical Officer	6 Nurses	5 Nurses *Medical Officer as needed from Inpatient Ward

^a^ Estimation taken from extrapolation of monthly registers from July 2019 site visits

^b^ https://www.ubos.org/onlinefiles/uploads/ubos/pdf%20documents/PNSD/2010MOHStatAbst.pdf and https://health.go.ug/affiliated-institutions/hospitals

^c^ Figures provided by EU staff during site visits in July 2019

#### Specifying the production process

In alignment with ongoing monitoring and evaluation by WHO and MoH, five sentinel conditions were selected by an expert panel as representative of conditions highly amenable to timely and appropriate EC ([21]; Reynolds TA, Pigoga JL, Adam H, Kalanzi J, Mirembe V, Sawe H, Wallis LA.: Assessing the impact of a low-cost WHO intervention package for emergency units in two hospitals in Uganda. 2020, Unpublished). Conditions were process mapped to identify equipment and supplies involved in the current practice of care. For each condition, a range of likeliest treatment pathways were first identified using procedures outlined in the AFEM 2nd edition Handbook [22] and later validated in discussions with global emergency care experts, Ugandan health care providers, and during visits to the hospital sites. This process resulted in the identification of 13 crucial interventions in the delivery of care for the five sentinel conditions. A table of these treatments processes can be found in the supplementary materials. Our expert panel identified a list of single use supplies (such as sundries and consumables), reusable supplies and capital inputs (such as medical equipment and devices), medication received, labour and diagnostic tests required for each intervention. Our approach in costing for care was to define the incremental cost associated with implementing EC described in the WHO EC Toolkit at existing hospitals. These hospitals were assumed to include some infrastructure capacities, such as building and utilities, which were not considered in our costs.

The sample population for the study was comprised of patients receiving unscheduled care for the treatment of five sentinel conditions; post-partum haemorrhage (PPH), road traffic incident (RTI), asthma, pneumonia, paediatric diarrhoea. Patients presenting to the unit with one of the selected conditions but whom did not receive one of the 13 identified treatments were excluded from the sample.

#### Enumerating inputs for each process

Each instance of resource utilization was counted at the individual patient level. Enumeration of inputs followed Hendricks et al. guidelines, using the bottom-up micro-costing method [23]. Study data were collected and managed using REDCap (Research Electronic Data Capture, a secure, web-based software platform designed to support data capture for research studies, hosted at the University of Cape Town [24].

All efforts were made to observe a minimum of 25 cases for each intervention, and data collectors were instructed to observe the same intervention at different points across the four-week period to ensure time-based data was representative of various providers. Data were largely collected from the emergency unit, although occasional cases of PPH and paediatric cases were captured in the Maternal and Children’s wards respectively. Six Ugandan data collectors were on site, daily, to observe all eligible cases. Data collectors identified potential cases as they arrived on site and followed providers throughout the duration of the delivery of intervention. During observation, data collectors noted all supplies, medicines and medical devices used throughout the intervention, as well as the occurrence of any diagnostic tests. The number of medical supplies used in the delivery of the interventions were tallied on a daily basis. Data clerks either entered data directly to REDCap using tablets, or first captured data on paper tools which were later uploaded into the online system.

Although services at government facilities are free of charge, anecdotal evidence suggest that due to extreme resource constraints patients may be asked to purchase their own medical supplies in order to receive timely care. To capture instances of out-of-pocket payment for single use items, medications and diagnostic tests, data collectors were asked to indicate if the item was paid for by the patient, presumably from a nearby pharmacy or private hospital or clinic.

Time-motion methodology was used to capture the resource use of labour [18]. Labour time was defined as hands-on time of providers in the delivery of an intervention, from the moment a provider decides on a course of action in treatment to the completion of that action. Tablet and phone timers were used to capture time spent in delivering care. Only activities and tasks which could be observed by the data clerk were captured, including diagnosis, stabilization and treatment of patients. In addition, data collectors captured the cadre of providers and number of each cadre present while delivering the intervention.

#### Costs

Unit costs of single use supplies, medicines and diagnostic tests were obtained from the National Medical Stores (NMS) price survey report, the primary provider of medical supplies to government hospitals [25]. The price of any items missing from the NMS report were obtained from Joint Medical Stores (JMS) – one of the largest private medical supply providers in the country. Estimated costs for diagnostics tests were taking from the literature [26–29]. Labour unit costs were expressed as cost per minute, calculated by dividing each cadre’s annual salary – obtained online from Ministry of Public Service salary structure for the fiscal year 2018–2019 – by the working minutes in each year (252 working days × 8 h per day × 60 mins per hour = 120,960) [30].

Reusable items, such as monitors and forceps, were distilled to a cost per each use. This was derived from the purchasing cost of each device, discounted at a rate of 3% over a useful lifespan appropriate to each item and divided by the presumed usage of once per day. On average, economic costs of capital items were 12% higher than accounting costs. Certain high use items, such as stethoscopes, were assumed to be used fifteen times per day based on expert opinion of in-country physicians and amended accordingly. All costs were converted from Ugandan Shillings to US Dollars at annual exchange average rate for 2017 (1 dollar = 3700 shilling) [20]. Resource use and unit costs attributable the interventions are listed in Table 2 (additional materials).

**Table 2:** Resource unit costs

Items	Unit	Cost per unit (Ugandan Shilling)	Cost per unit (USD)	Source
**Labour**				
Nursing Student (U8)	minute	22	0.01	FY 2018–19 Salary Structure [29]
Nurse (U5)	minute	79	0.02	FY 2018–19 Salary Structure [29]
Interns (U5)	minute	79	0.02	FY 2018–19 Salary Structure [29]
Medical Officer (U4- Med 1)	minute	292	0.08	FY 2018–19 Salary Structure [29]
Clinical Officer (U4- Med 2)	minute	93	0.03	FY 2018–19 Salary Structure [29]
**Single use supplies**				
Adhesive tape	roll	39,183	10.59	Joint Medical Stores
Endotracheal tube 3MM	each	1300	0.35	National Medical Stores [24]
Alcohol swabs	each	113.53	0.03	Estimate
Antiseptic solution (iodine)	200 ml	3240	0.88	National Medical Stores [24]
Nebulizer mask	each	38,394	10.38	Joint Medical Stores
Cotton roll	500 g roll	8590	2.32	National Medical Stores [24]
Compression bandage	each	1412	0.38	National Medical Stores [24]
Non sterile gloves	pair	105.53	0.03	National Medical Stores [24]
Sterile gloves	pair	691.1	0.19	National Medical Stores [24]
Gauze dressing	90 cm × 90 m	41,346	11.17	National Medical Stores [24]
Crepe bandage 4in	roll	954	0.26	National Medical Stores [24]
Crepe bandage 6in	roll	1702	0.46	National Medical Stores [24]
Blood transfusion giving set	each	615	0.17	National Medical Stores [24]
IV giving set	each	502.93	0.14	National Medical Stores [24]
Suction catheter (FG 16)	each	361	0.10	National Medical Stores [24]
Non adhesive dressing	90 cm x91m	36,492	9.86	National Medical Stores [24]
Plaster of Paris 6in	roll	3559	0.96	National Medical Stores [24]
Plaster of Paris 8in	roll	3559	0.96	National Medical Stores [24]
Gauze roll	roll	783.75	0.21	National Medical Stores [24]
Nonrebreather mask	each	12,199	3.30	Joint Medical Stores
Lubricant (KY jelly)	42 g tube	3770	1.02	National Medical Stores [24]
NG tube size 10	each	908	0.25	National Medical Stores [24]
NG tube size 14	each	489	0.13	National Medical Stores [24]
NG tube size 16	each	454	0.12	National Medical Stores [24]
Average NG tube	each	617	0.17	National Medical Stores [24]
Tongue depressor	each	50	0.01	National Medical Stores [24]
Water for injection	10 ml vial	80	0.02	National Medical Stores [24]
Surgical blade average	each	237.81	0.06	National Medical Stores [24]
Average IV Cannula	each	366.29	0.10	National Medical Stores [24]
Average Syringe	each	232.83	0.06	National Medical Stores [24]
**Reusable supplies**	***Useful life years***	***Economic costs (accounting costs)***		
Cervical collar	5	53.87 (49.35)	0.01 (0.01)	Joint Medical Stores
Bucket	2	13.25 (12.67)	0.004 (0.003)	Joint Medical Stores
Artery forceps	7	6.08 (6.06)	0002 (0.002)	Joint Medical Stores
Needle holder	7	4.12 (3.67)	0.001 (0.001)	Joint Medical Stores
Laryngoscope	7	42.34 (37.69)	0.01 (0.01)	Joint Medical Stores
Kidney dish	10	2.44 (2.08)	0.001 (0.001)	Joint Medical Stores
Bag valve mask	5	30.50 (27.94)	0.01 (0.01)	Joint Medical Stores
O2 cylinder	5	11.96 (10.96)	0.003 (0.003)	National Medical Stores [24]
Scalpels	5	0.07 (0.07)	0.00002 (0.00002)	National Medical Stores [24]
Scissors	10	2.75 (2.34)	0.001 (0.001)	Joint Medical Stores
O2 concentrator	10	638.22 (544.40)	0.17 (0.15)	Joint Medical Stores
Thermometer	10	1.50 (1.28)	0.0004 (0.00)	Joint Medical Stores
ECG	7	1007.41 (896.59)	0.27 (0.24)	Joint Medical Stores
Nebulizer machine	10	146.98 (125.38)	0.04 (0.03)	Joint Medical Stores
Mechanical ventilator	7	12,136.33 (10,801.34)	3.28 (2.92)	Joint Medical Stores
Stethoscope	5	13.64 (12.49)	0.004 (0.003)	Joint Medical Stores
Blood pressure cuff	2	31.03 (29.68)	0.008 (0.008)	Joint Medical Stores
IV infusion pump	10	589.03 (502.44)	0.16 (0.14)	Joint Medical Stores
Cardiac monitor	10	361 (307.74)	0.09 (0.08)	Joint Medical Stores
Glucometer	7	23.75 (21.14)	0.006 (0.01)	Joint Medical Stores
Vital signs monitor	10	361 (307.74)	0.09 (0.08)	Joint Medical Stores
Defibrillator	7	3089.85 (2749.97)	0.84 (0.74)	Joint Medical Stores
Portable ultrasound	10	8497.48 (7248.35)	2.30 (1.96)	Joint Medical Stores
Pulse Oximeter	7	767.50 (683.08)	0.21 (0.18)	Joint Medical Stores
Suction device	10	456.75 (389.61)	0.12 (0.11)	Joint Medical Stores
**Medicines**	***Unit***			
Iodine	200 ml	3240	0.88	National Medical Stores [24]
Misoprostol	200mcg	360.08	0.10	National Medical Stores [24]
Oxytocin	10 IU	194.05	0.05	National Medical Stores [24]
Tranexamic acid	500 mg	3600	0.97	Joint Medical Stores
Diazepam	5 mg tab	4.5	0.00	National Medical Stores [24]
Hydrocortisone	100 mg	1224.32	0.33	National Medical Stores [24]
Salbutamol	2.5 ml vial	2559.8	0.69	National Medical Stores [24]
Aminophylline	250 mg/10 ml	700	0.19	National Medical Stores [24]
Atropine	1 mg/1 ml	126.62	0.03	National Medical Stores [24]
Diazepam Injection	10 mg/2 ml	296.16	0.08	National Medical Stores [24]
Diclofenac Tab	tab	9.58	0.00	National Medical Stores [24]
Diclofenac injection	75 mg/3 ml	117.72	0.03	National Medical Stores [24]
Bupivacaine	4 ml amp	5940	1.61	National Medical Stores [24]
Trap	325mcg tab	323.78	0.09	Joint Medical Stores
Zinc sulphite tablet	20 mg tablet	31.2	0.01	National Medical Stores [24]
Lidocaine injection	5 ml	2318	0.63	National Medical Stores [24]
Lignocaine injection	20 ml	3536	0.96	National Medical Stores [24]
Misoprostol tablet	200mcg	255.54	0.07	National Medical Stores [24]
Paracetamol tablets	500 mg tab	11.5	0.00	National Medical Stores [24]
Paracetamol syrup	125 mg	1363	0.37	National Medical Stores [24]
Paracetamol vial	100 ml	7233	1.95	Joint Medical Stores
Paracetamol suppository	125 mg	960	0.26	National Medical Stores [24]
Tramadol ampoule	100 mg/2 ml	897.8	0.24	National Medical Stores [24]
Tolfree	100mcg tab	348	0.09	Joint Medical Stores
Pethidine	100 mg/2 ml	2257.8	0.61	National Medical Stores [24]
Ampiclox tab	250 mg	95	0.03	National Medical Stores [24]
Ampiclox IV	250 mg	634.43	0.17	National Medical Stores [24]
Ampicillin	500 mg	376.12	0.10	National Medical Stores [24]
Aminophylline IV	250 mg/10 ml	700	0.19	Joint Medical Stores
Amoxycillin tab	tab	78	0.02	Joint Medical Stores
Amoxycillin syrup	100 ml	3765	1.02	National Medical Stores [24]
Ascoril Syrup	100 ml	5033	1.36	National Medical Stores [24]
Azithromycin tab	500 mg	614.67	0.17	National Medical Stores [24]
Cloxacillin IV	500 mg	396.88	0.11	National Medical Stores [24]
Cefotaxime	1 g	11,971	3.24	National Medical Stores [24]
Ceftriaxone	1 g	973	0.26	National Medical Stores [24]
Cefixime tabs	200 mg	1576	0.43	Joint Medical Stores
Cefixime syrup	50 mg/5 ml	1200	0.32	Joint Medical Stores
Ciprofloxacin tab	500 mg	84.79	0.02	National Medical Stores [24]
Folic Acid pill	5 mg	500	0.14	National Medical Stores [24]
Gentamycin	80 mg/2 ml	127.44	0.03	National Medical Stores [24]
Hydrocortisone	100 mg	1224.32	0.33	National Medical Stores [24]
Ibuprofen Syrup		1257	0.34	National Medical Stores [24]
Metronidazole tab	200 mg	13.5	0.00	National Medical Stores [24]
Metronidazole IV	500 mg/100 ml	794	0.21	National Medical Stores [24]
Metronidazole Suspension	100 mg	1322	0.36	National Medical Stores [24]
Penicillin	600 mg	270	0.07	National Medical Stores [24]
Phenobarbiton	30 mg tab	32.22	0.01	Joint Medical Stores
D5	250 ml	137.5	0.04	National Medical Stores [24]
D10	250 ml	1200	0.32	Joint Medical Stores
D50	100 ml	1397	0.38	National Medical Stores [24]
Dextrose	5% 500 ml	1375	0.37	National Medical Stores [24]
Fenobabitone (Phenobarbital)	200 mg/2 ml	12,100	3.27	National Medical Stores [24]
Manitol	100 ml	2800	0.76	National Medical Stores [24]
Normal saline	250 ml	1100	0.30	National Medical Stores [24]
Ringers Lactate	250 ml	1000	0.27	National Medical Stores [24]
Tranexamic acid	500 mg inj	3600	0.97	Joint Medical Stores
**Diagnostic tests**				
Complete Blood Count		14,763	3.99	Amukele et al. [25]
Malaria test		6253	1.69	Schroeder et al. [26]
Stool		8880	2.40	Schroeder et al. [26]
Urine sample		9657	2.61	Schroeder et al. [26]
Sputum		7770	2.10	Whitelaw et al. [27]
Gene Xpert		77,700	21.00	Hsiang et al. [28]
Cross matching		9324	2.52	Schroeder et al. [26]
Typhoid test		12,543	3.39	Schroeder et al. [26]
Ultrasound		15,000	4.05	Expert estimation
X-ray		10,000	2.70	Expert estimation
CT Scan		200,000	54.05	Expert estimation

The following items were included in calculating the total direct costs; personnel time, equipment/reusable supplies, single use supplies, diagnostic tests and medications (Supplementary materials). A series of assumptions were made in our analytical approach to costing these services. Costs were calculated at patient level by multiplying the frequency of use for each item used by the unit price, added with the time spent conducting the intervention by staff multiplied by labour costs per minute, to determine the total cost. Costs were apportioned to each intervention by the average number of times consumables and drugs were utilized for each intervention. Capital costs included all reusable items and medical devices. Cost per use of reusable equipment was apportioned to each intervention by the average number of times utilized for that intervention. Salaries were proportioned by the average amount of time spent for that intervention by the care team. A table summarizing details of all sources of information, basic analysis and cost share assumption per costing area is provided in Table 3. Data was analysed for measures of central tendency for each condition and each intervention using Microsoft Excel (Microsoft Redmond, WA), and STATA 13 software (StataCorp. 2013. Stata Statistical Software: Release 13. College Station, TX: StataCorp LP). A Kruskal-Wallis H test was conducted to determine if total cost was different for the sentinel conditions. A post-hoc Nemenyi test was performed to make pairwise comparisons between the conditions.

**Table 3:** Costing and cost share assumptions

Costing area	Sources of Information	Basic Analysis	Cost share assumptions
Consumables (*Price and Quantity*)	***Quantity***: observed by on-site data collectors ***Prices:*** Market price from government distributors and where necessary other local distributors .	The expenditure on consumables was calculated from the quantity used multiplied by the unit prices.	Costs were apportioned to each intervention by average number of times utilizing the consumables for each intervention.
Medicines and Drugs (*Price and Quantity*)	***Price:*** Market price lists were obtained from government distributors and where necessary other local distributors. ***Quantity:*** Observed by on-site data collectors	The amount spent on drugs and medicines were calculated by multiplying quantity used and unit price for each drug or medicine.	Costs were apportioned to each intervention by average number of times utilizing these drugs for each intervention.
Reusable equipment and machinery (*Quantity, Price and Average life*)	***Quantity:*** Observed by on-site data collectors ***Price:*** Market price from government distributors, local distributors and relevant websites. ***Average life:*** literature review, interviews with staff at health facility	Cost of equipment and machinery were distilled to a cost per use. The onetime costs of purchase of reusable equipment and machinery were annualized for their average life using a discount rate of 3% and then divided by number of uses per year.	The cost per use was then apportioned to each intervention by the average number of times utilized for that intervention.
Salaries of human resource *(Time and Salary)*	***Salary:*** Public Service salary structure for the fiscal year 2018–2019 ***Time:*** Observed to the nearest minute by data on-site data collectors.	Annual salaries of the health staff of the facility were distilled to an per minute unit cost by dividing annual salary by number of working minutes in a year (120,960) Amount spent on human resources was calculated by multiplying number of minutes in care by price for one minute of provider time.	Proportioned by the average amount of time spent for that intervention by the care team.

## Results

Eight hundred seventy-two cases across the five sites were captured for this study. The distribution of the cases by condition and intervention, as well as the median costs of treatment, are summarised in Table 4. RTI is the most common presentation observed across hospital sites accounting for over 42% of all cases captured. Delivery of IV fluids was the most commonly observed intervention (20.41%), followed by delivery of antibiotics (18.35%), analgesia (11%) and oral rehydration (10.4%). The overall median (IQR) cost of care across all conditions is $15.53 (14.44 to 19.22). The median (IQR) cost per condition was highest for PPH at $17.25(15.02 to 21.36), followed by $15.96(14.51 to 20.30) for RTI, $15.90(14.76 to19.30) for asthma, $15.55(14.65 to 20.12) for pneumonia, and $14.61(13.74 to15.57) for paediatric diarrhoea. Cost of care for a patient with RTI varied the most significantly, with a mean (SD) of $21.59(25.14) and median (IQR) $16.05(14.51 to 20.30) as compared to paediatric diarrhoea which varies the least, with mean (SD) $15.74(3.46) and median (IQR) $14.65(13.74 to 15.57). The median cost per intervention was highest for fracture reduction & splinting ($27.77), wound closure ($18.76) and haemorrhage control ($18.07). Cost of care per case for these interventions varied the most significantly, fracture reduction and splinting with a mean (SD) of $38.23(56.93) and median (IQR) $27.77(22.00 to 31.50) as compared to paediatric diarrhoea which varies the least, with mean (SD) $15.74(3.46) and median (IQR) $14.65(13.74 to 15.57). A Krauskal-Wallis test yielded statistically significant difference in cost values between sentinel conditions H = 92.92, *p* = 3.15E-19. At a *P* value of < .05, the post-hoc Nemenyi test revealed paediatric diarrhoea has a statistically significant lower median cost compared to all other conditions but did not yield any significant differences in median cost between the remaining four sentinel conditions (Table 5).

**Table 4:** Cost of care per sentinel condition and intervention in 2017 US Dollars

	n	Median (IQR)	Mean (SD)	Min	Max	Kruskal Wallis H
**TOTALS**	**872**	**15.53 (14.44–19.22)**	**19.03 (16.96)**	**13.66**	**413.11**	
***Cost in USD by sentinel condition***						
RTI	371	15.96 (14.51–20.30)	21.59 (25.14)	13.71	413.11	H = 92.92
PPH	59	17.25 (15.02–21.36)	19.97 (9.10)	13.75	73.79	*p* < .0001
Asthma	66	15.90 (14.76–19.30)	17.49 (3.67)	13.70	28.01	*n* = 872
Pneumonia	186	15.55 (14.65–20.12)	15.55 (4.39)	13.66	38.26	
Paediatric diarrhoea	190	14.61 (13.74–15.57)	15.74 (3.46)	13.66	30.31	
***Cost in USD by intervention***						
Wound closure	69	18.76 (16.63–21.79)	20.71 (8.51)	14.18	73.54	
Fracture reduction & splinting	55	27.77 (22.00–31.50)	38.2 (56.93)	14.43	413.11	
Haemorrhage Control	56	18.07 (17.10–22.83)	25.55 (19.48)	13.94	83.94	
IV fluids	178	15.79 (15.05–17.85)	18.42 (8.54)	13.96	83.89	
Oral Rehydration	91	13.74 (13.70–13.83)	14.12 (1.42)	13.66	23.57	
Antibiotics	160	15.44 (14.74–18.0)	16.92 (3.49)	13.89	38.26	
Oxygen	80	14.69 (13.91–20.00)	16.99 (4.01)	13.70	29.68	
Nebulisation	39	15.84 (15.15–18.64)	17.36 (3.50)	13.98	28.01	
Analgesia	96	14.22 (13.96–14.51)	14.71 (2.58)	13.66	37.72	
Transfusion/blood given	33	15.54 (14.68–18.31)	18.04 (9.75)	14.01	71.20	
Oral or Nasal Pharyngeal Airway^a^	2	16.73 (16.69–16.77)	16.73 (0.12)	16.64	16.82	
Needle decompression^a^	6	15.76 (15.60–16.02)	15.76 (0.50)	15.27	16.72	
Intubation^a^	7	29.31 (17.45–35.86)	27.08 (10.19)	15.24	38.42	

^a^Small sample size

**Table 5:** Correlation results matrix of Nemenyi test of sentinel conditions

	RTI	PPH	Asthma	Pneumonia	Paediatric diarrhoea
RTI	–	*q-stat* = 2.374 *p* = 0.448	*q-stat* = 0.260 *p* = 0.999	*q-stat* = 2.012 *p* = 0.613	*q-stat* = 12.381 *p* < 0.001^a^
PPH		–	*q-stat* = 2.051 *p* = 0.595	*q-stat* = 3.437 *p* = 0.109	*q-stat* = 9.644 *p* < 0.001^a^
Asthma			–	*q-stat* = 1.019 *p* = 0.952	*q-stat* = 7.487 *p* < 0.001^a^
Pneumonia				–	*q-stat* = 8.956 *p* < 0.001^a^
Paediatric diarrhoea					–

^a^significant at the 0.05 level, q-crit 3.875

Single use supplies contributed considerably to the total cost of care (67%), followed by reusable supplies (16%) and diagnostic tests (8%). Medications (5%) and labour (4%) were the least significant drivers of cost (Table 6). This similar distribution pattern held true across most conditions and interventions with the exception of haemorrhage control, where labour costs contributed to 22% of total costs.

**Table 6:** Components of direct cost of care per sentinel condition and intervention

	Single Use Supplies	Reusable Supplies	Medications	Labour	Diagnostics
**TOTALS**	**67%**	**16%**	**5%**	**4%**	**8%**
***Proportion of total cost by sentinel condition***					
RTI	69%	14%	5%	4%	8%
PPH	64%	15%	8%	7%	6%
Asthma	62%	18%	6%	6%	8%
Pneumonia	64%	18%	3%	4%	11%
Paediatric diarrhoea	70%	19%	3%	3%	4%
***Proportion of total cost by intervention***					
Wound closure	69%	15%	3%	7%	6%
Fracture reduction & splinting	76%	8%	7%	5%	3%
Haemorrhage Control	57%	12%	5%	4%	22%
IV fluids	62%	17%	9%	4%	8%
Oral Rehydration	76%	21%	0%	1%	2%
Antibiotics	68%	18%	2%	3%	8%
Oxygen	64%	19%	1%	6%	10%
Nebulisation	62%	18%	9%	5%	6%
Analgesia	75%	21%	3%	1%	1%
Transfusion/blood given	63%	17%	0%	5%	15%
Oral or Nasal Pharyngeal Airway^f^	64%	18%	0%	17%	0%
Needle decompression^f^	70%	19%	0%	11%	0%
Intubation^f^	55%	12%	1%	22%	10%

^a^ Single use supplies includes all supplies and equipment which are discarded after one use such as gauze

^b^ Reusable supplies include all supplies and equipment which are used more than once such as stethoscopes, monitors and kidney dishes

^c^ Medications includes all drugs used in treatment

^d^ Labour includes all observed time of care delivered by health care workers

^e^ Diagnostics include all tests and procedures used in identification and treatment such as, radiology and labs

^f^Small sample size, therefore results unreliable at intervention level

Median labour time and proportional time of care by cadres is presented in Table 7. Providers spent the most time in care for patients with asthma 24.5 min (10 to 69) and PPH at 23 min (9 to 46), and the least time on interventions with patients with paediatric diarrhoea at 8 min (3 to 25). Labour time was highest for the intervention of fracture reduction & splinting at 42.5 min (21.75 to 64.25) and wound closure at 39.5 min (25 to 64). RTI care had a higher proportion of medical specialist time than any other condition (12%). Care for paediatric diarrhoea was almost exclusively delivered by nurses (90%). 64% of labour was provided was provided by nurses or nursing student, which is in alignment with the literature regarding delivery of care in sub-Saharan Africa [31].

**Table 7:** Median time spent by providers and distribution by cadre

	Time Median (IQR) in minutes	Proportion of total time specialist care^a^	Proportion of total time nurse care^b^	Proportion of total time medical care^c^
**TOTALS**	**15 (5–45)**	**2%**	**64%**	**30%**
***By condition***				
RTI	18 (7–42)	12%	53%	35%
PPH	23 (9–46)	0%	56%	43%
Asthma	24.5 (10–69)	0%	61%	38%
Pneumonia	15 (5–70)	1%	72%	26%
Paediatric diarrhoea	8 (3–25)	0%	90%	9%
***By intervention***				
Would closure	39.5 (25–64)	2%	48%	39%
Fracture reduction & splinting	42.5 (21.75–64.25)	12%	49%	23%
Haemorrhage Control	22.5 (9.5–58.5)	0%	54%	41%
IV fluids	20 (10.25–43.5)	1%	80%	18%
Oral Rehydration	3 (2–5)	0%	92%	7%
Antibiotics	14 (8–38)	0%	85%	14%
Oxygen	15 (4–73.25)	0%	52%	45%
Nebulisation	25 (11–63)	0%	64%	36%
Intubation^d^	128 (68.5–295)	0%	46%	52%
Analgesia	5 (1–9.25)	4%	82%	13%
Transfusion/blood given	25 (8–45)	0%	57%	41%
Oral or Nasal Pharyngeal Airway^d^	83 (80–86)	0%	0%	92%
Needle decompression^d^	22 (16.25–25.5)	0%	0%	89%

^a^ Specialist care includes all specialists including: surgical, paediatric, obstetrics, internal medicine and other

^b^ Nurse care includes nursing students and nurses

^c^ Medical care includes interns, medical officers and clinical officers

^d^Small sample size, therefore results unreliable at intervention level

Most supplies, medications and screening tests were provided by the hospital (94%). Only 6% of the 5490 items captured were paid for by patients. Over 50% of all screening tests (Ultrasound, X-ray, CT Scan) were paid for by patients as presented in Table 8.

**Table 8:** Proportion of out-of-pocket payment by patients for screening tests

	Ultrasound	X-ray	CT Scan	Other^a^
**TOTALS (N)**	**1**	**79**	**9**	**123**
Hospital provided (n)	0	39	0	89
Out of pocket by patient (n)	1	40	9	34
% out of pocket by patient	100%	51%	100%	28%

^a^Other includes complete blood counts, urine, stool and sputum tests

## Discussion

This study is the first to describe the direct costs of EC in hospitals in Uganda by observing the delivery of clinical services, using robust activity-based costing and time motion methodology. The costs for EC interventions are found to be considerably lower than other health programmes in the Ugandan setting such as tuberculosis treatment ($151), HIV anti-retroviral therapy ($628) and obstetric fistula repair ($378) [32–34]. Emergency care could be highly impactful in Uganda, where injuries from trauma and road traffic incidents are a leading cause of death [35], accounting for 7% of all mortality in public health facilities in 2017 to 2018 [36].

Our methodology attempted to capture all relevant costs for implementing WHO recommended care delivery in the emergency care setting. Our analysis finds that single use supplies, as opposed to multiuse supplies, are the most important cost drivers to the total cost of EC. Reusable resources which have a cost of use and are broadly less sensitive to variations in patient load, such as reusable medical equipment and labour, contributed significantly less to the total cost. Discounting of capital costs resulted in very minimal changes to results with median economic costs being 2.4% higher than median accounting costs. This finding implies that marginal costs contribute sizeably to the total cost of EC care; as such, economies of scale – where unit cost decreases as the volume of output increases – may not be as significant to EC care interventions as previously suggested [37]. This finding aligns with current evidence of the higher than expected marginal costs of outpatient EC in HIC [12].

These findings hint that a further cost saving may be realized if additional reuse of supplies is promoted where reasonable. To recommend such an approach, the impact on effectiveness of patient care, which falls outside of the scope of this work, would need to be better understood. In many ways this practice of reuse is already routine in LMIC settings where supplies such as suturing tools, disposed after a single use in HIC settings, are commonly sterilised and reused in LMICs. During our study we observed the use of cardboard boxes for splints or gloves as tourniquets, further demonstrating the extreme resourcefulness of practitioners in this setting. In the absence of EC guidelines and recommendations many hospitals have improvised walls made of tarps, and IV bags zip-tied to walls rather than hanging from drip stands. Future research should endeavour to better understand the impact which operating under resource scarcity has on the delivery of EC and how local adaptations may provide further cost-savings.

Although we suspected that patients would be responsible to pay for many supply items if unstocked, from our limited data we found little evidence of this practice and low frequencies of out-of-pocket payment made by patients. On the other hand, 100% of CT Scans and Ultrasounds and over 50% of X-Rays were paid for by patients, likely because almost no sites had CT Scanners at their facility, and many had limited x-ray machines or film. This pattern indicates particular challenges in diagnostic testing resources and access which could interrupt patient care.

Assessing the economic cost of EC is complex because of the heterogenous nature of EC conditions and their wide-ranging clinical features. Inconsistent cost data further convolutes the sparsity of information regarding EC in LMICs. In South Africa, a $6749 difference was observed between a top-down and bottom-up costing approach of the average healthcare cost per road traffic injury patient [38]. While efforts have been made to quantify the pattern of resource use and procedure utilization in functional emergency units in Uganda, the actual cost of delivering these services in this setting remains unclear [16]. As a result, we rather unconventionally approach the costing of EC services by presenting the cost of care for five sentinel conditions, as well as the cost per intervention conducted. We predicted finding a high variation in cost for each condition. Interestingly, our results show that the cost of delivering care is relatively stable across conditions, and only statistically lower for paediatric diarrhoea. As expected, costs of RTIs had the highest variability in cost, due to the wide range of injuries which fall under that condition; but overall RTI interventions do not have significantly higher costs than other conditions (except paediatric diarrhoea). This finding suggests that there may be a set of basic, low-cost, high impact, lifesaving approaches and interventions such as IV support or blood transfusion, which provide significant value for money. Benefits of economies of scope—where unit cost of production decrease as variety of products increase--may still apply to extending EC services in the context of existing health systems [39].

Our time-motion study found that nurses provided most of the labour for all interventions, which is supported by their representation of 75% of the healthcare workforce [40]. This pattern touches on the significance of the nursing workforce in the delivery of emergency care in LMICs, which may impact the planning of training and labour budgets for delivering EC, and aligns with broader discussions of task-shifting acute care to nursing cadres [41].

This study illuminates some of the key challenges to pursuing a research agenda in cost-effectiveness research for EC in LMICs. Collecting rigorous data is costly and time consuming, and in contexts where care delivery may be fractured it can be challenging to identify the emergency centre’s contributions to both the budget and patient survival of a hospital. As such, strengthening routine data collection systems to better understand supply use in public facilities could greatly aid future economic analyses and cost-effectiveness studies. Further research to better understand the impact creative material use and reuse may have on practice and budgets in a resource limited setting is also essential. Through these findings we can begin to understand what it means to operate EC services under resource scarcity.

### Limitations

This study has potential limitations. First, free text entry of data by non-medically trained research assistants resulted in a small amount of data (0.5%) regarding medications being lost due to misspelling, incorrect transcription or unidentified unit measurements. Second, a widespread lack of basic equipment and medicines for EC has been well categorized in Uganda [14] and treatment decisions and resource used in the care of patients may be influenced by this scarcity. In order to appropriately represent the context, we adopted a direct observation approach using activity-based costing and time motion methodology. As a result, while accurately reflecting the actual costs of care, our findings may underestimate the costs of ideal care that is uncompromised by persistent resource limitation. Future research could examine the impact of resource scarcity on care delivery behaviour. Similarly, the full costs of EC are likely underestimated, as indirect costs are not captured in this study due to resource limitations. Nevertheless, this study provides detailed direct cost information that can inform future research which aims to consider indirect costs such as social opportunity costs and patient and caretaker time.

Additionally, restocking at hospitals occurs on a monthly basis and may impact the supply use patterns of providers where patients at the beginning of the month receive more services, or medicines and supplies are reserved for high-risk patients. To account for these variations data were collected for a full period of a month but the impacts of inventory upon patterns of resource use fall beyond the scope of this paper and should be prioritized in future research. Certain interventions, such as intubation, oral or nasal pharyngeal airway insertion and needle decompression, are extraordinarily rare in the Ugandan setting likely due to the absence of the next step in the chain of care such as intensive care units. Therefore, these interventions are under-powered in our findings. We included these interventions in our aggregated analysis to further shed light on EC healthcare utilizations but were unable to draw any conclusions at the intervention level given the small sample size.

Despite best efforts to move towards formalized EC in Uganda, there is still much to be done to systematize the approach to EC. Staffing, equipment and physical space differ greatly between hospitals. Due to the unique, country-specific characteristics of Uganda, the data and findings here reflect site-specific practices with specific patient populations within the particulars of the public Ugandan health care system and local prices. Therefore the results of our study are not generalizable to other LMICs.

## Conclusion

This study finds that EC in Uganda is managed at considerably lower costs compared to many priority health interventions in low-resource settings. Given the prevalence of these conditions and our research findings, it is likely that allocating resources to strengthening EC may be an extremely valuable investment to the health system. The results provide evidence on the direct costs of delivering EC in LMICs, which can be further used to estimate the health care costs faced by LMICs looking to expand access to EC services. Further research assessing acute care delivery and the impact of creative material use and reuse in resource scarce settings would be useful in planning wider health care delivery systems development.

## Supplementary Information

**Additional file 1: Supplementary Table 1.** Treatment processes for five sentinel conditions.

## Data Availability

The datasets used and/or analysed during the current study are available on reasonable request. The corresponding author [KW] can work with interested researchers to secure approval from the relevant authorities (Ministry of Health) to reuse the dataset for research.

## Abbreviations

EC: Emergency Care
HIC: High income countries
LMIC: Low-and middle income countries
MoH: Ministry of Health
PPH: Post-partum haemorrhage
RRH: Regional Referral Hospital
RTI: Road traffic incident
USD: United States Dollar
WHO: World Health Organisation

## References

[1] MacKenzie EJ, Rivara FP, Jurkovich GJ, Nathens AB, Frey KP, Egleston BL (2006). A National Evaluation of the Effect of Trauma-Center Care on Mortality. NEJM.

[2] Roudsari BS, Nathens AB, Arreola-Risa C, Cameron P, Civil I, Grigoriou G (2007). Emergency medical service (EMS) systems in developed and developing countries. Injury..

[3] Peleg K, Aharonson-Daniel L, Stein M, Kluger Y, Michaelson M, Rivkind A (2004). Increased survival among severe trauma patients: the impact of a national trauma system. Arch Surg.

[4] Nathans AB, Jurkovich GJ, Rivara FP, Maier RV (2000). Effectiveness of state trauma Systems in Reducing Injury-Related Mortality: a National Evaluation. J Trauma Acute Care Surg.

[5] Razzak J, Usmani MF, Bhutta ZA (2019). Global, regional and national burden of emergency medical diseases using specific emergency disease indicators: analysis of the 2015 global burden of disease study. BMJ Glob Heal.

[6] Roth GA, Abate D, Abate KH, Abay SM, Abbafati C, Abbasi N (2018). Global, regional, and national age-sex-specific mortality for 282 causes of death in 195 countries and territories, 1980–2017: a systematic analysis for the global burden of disease study 2017. Lancet..

[7] Chang CY, Abujaber S, Reynolds TA, Camargo CA, Obermeyer Z (2016). Burden of emergency conditions and emergency care usage: new estimates from 40 countries. Emerg Med J.

[8] Obermeyer Z, Abujaber S, Makar M, Stoll S, Kayden SR, Wallis LA (2015). Emergency care in 59 low- and middle-income countries: a systematic review. Bull World Health Organ.

[9] Razzak JA, Kellermann AL (2002). Emergency medical care in developing countries: is it worthwhile?. Bull World Health Organ.

[10] Reynolds TA, Stewart B, Drewett I, Salerno S, Sawe HR, Toroyan T (2017). The Impact of Trauma Care Systems in Low- and Middle-Income Countries. Annu Rev Public Health.

[11] Werner K, Risko N, Burkholder T, Munge K, Wallis L, Reynolds T (2020). Cost–effectiveness of emergency care interventions in low and middle-income countries: a systematic review. Bull World Health Organ.

[12] Bamezai A, Melnick G, Nawathe A (2005). The cost of an emergency department visit and its relationship to emergency department volume. Ann Emerg Med.

[13] Stanford R, McLaughlin T, Okamoto LJ (1999). The cost of asthma in the emergency department and hospital. Am J Respir Crit Care Med.

[14] Ningwa A, Muni K, Oporia F, Kalanzi J, Zziwa EB, Biribawa C, et al. The state of emergency medical services and acute health Facility Care in Uganda: findings from a National Cross-Sectional Survey. Research Square. 2019. Available from:. 10.21203/rs.2.10966/v1. 10.1186/s12913-020-05508-8 PMC7346654 32646519

[15] Wesson HKH, Boikhutso N, Bachani AM, Hofman KJ, Hyder AA (2013). The cost of injury and trauma care in low-and middle-income countries: a review of economic evidence. Health Policy Plan.

[16] Bitter CC, Rice B, Periyanayagam U, Dreifuss B, Hammerstedt H, Nelson SW (2018). What resources are used in emergency departments in rural sub-Saharan Africa? A retrospective analysis of patient care in a district-level hospital in Uganda. BMJ Open.

[17] Adam H, Reynolds T, Officer SH (2019). Improving emergency care in Uganda. Bull World Health Organ.

[18] Frick KD (2009). Microcosting quantity data collection methods. Med Care.

[19] Cunnama L, Sinanovic E, Ramma L, Foster N, Berrie L, Stevens W (2016). Using top-down and bottom-up costing approaches in LMICs: the case for using both to assess the incremental costs of new Technologies at Scale. Health Econ.

[20] xe Historical Exchange Rates. [Internet] Available from: xe.com. Accessed 21st Feb 2020.

[21] Kivelehan S, Dixon J, Kalanzi J, Sawe H, Chien E, Robert J, et al. Impact of the WHO Basic Emergency Care Course in Tanzania and Uganda. [Preprint] 2019. Available from: 10.21203/rs.2.19074/v1.

[22] Wallis L, Checkett K, Reynolds T (2018). AFEM handbook of acute and emergency care.

[23] Hendriks ME, Kundu P, Boers AC, Bolarinwa OA, Te Pas MJ, Akande TM (2014). Step-by-step guideline for disease-specific costing studies in low- and middle-income countries: a mixed methodology. Glob Health Action.

[24] Harris PA, Taylor R, Thielke R, Payne J, Gonzalez N, Conde JG (2009). Research electronic data capture (REDCap) - a metadata-driven methodology and workflow process for providing translational research informatics support. J Biomed Inf.

[25] NMS (2017). 6th edition of the price survey report.

[26] Amukele TK, Jones R, Elbireer A (2018). Test cost and test accuracy in clinical Laboratories in Kampala, Uganda. Am J Clin Pathol.

[27] Schroeder LF, Elbireer A, Jackson JB, Amukele TK (2015). Laboratory diagnostics market in East Africa: a survey of test types, test availability, and test prices in Kampala, Uganda. PLoS One.

[28] Whitelaw A, Peter J, Sohn H, Viljoen D, Theron G, Badri M (2011). Comparative cost and performance of light-emitting diode microscopy in HIV-tuberculosis-co-infected patients. Eur Respir J.

[29] Hsiang E, Little KM, Haguma P, Hanrahan CF, Katamba A, Cattamanchi A (2016). Higher cost of implementing Xpert® MTB/RIF in Ugandan peripheral settings: Implications for cost-effectiveness. Int J Tuberc Lung Dis.

[30] Ministry of Public Service (2018). Salary Structure FY 2018/19 Schedule.

[31] Ugochukwu CG, Uys LR, Karani AK, Okoronkwo IL, Diop BN (2013). Roles of nurses in sub-Saharan African region. Int J Nurs Midwifery.

[32] Okello D, Floyd K, Adatu F, Odeke R, Gargioni G (2003). Cost and cost-effectiveness of community-based care for tuberculosis patients in rural Uganda. Int J Tuberc lung Dis Off J Int Union against Tuberc Lung Dis.

[33] Jain V, Chang W, Byonanebye DM, Owaraganise A, Twinomuhwezi E, Amanyire G (2015). Estimated costs for delivery of HIV antiretroviral therapy to individuals with CD4+ T-cell counts >350 cells/uL in rural Uganda. PLoS One.

[34] Epiu I, Alia G, Mukisa J, Tavrow P, Lamorde M, Kuznik A (2018). Estimating the cost and cost-effectiveness for obstetric fistula repair in hospitals in Uganda: a low income country. Health Policy Plan.

[35] Kimuli Balikuddembe J, Ardalan A, Khorasani-Zavareh D (2017). Nejati Kasiima Stephen Munanura a. road traffic incidents in Uganda: a systematic review of a five-year trend. J Inj Violence Res.

[36] Ministry of Health Republic of Uganda. Annual Health Sector Performance Report Financial Year 2017/18. 2017.

[37] Blank JLT, van Hulst BL, Valdmanis VG (2017). Concentrating Emergency Rooms: Penny-Wise and Pound-Foolish? An Empirical Research on Scale Economies and Chain Economies in Emergency Rooms in Dutch Hospitals. Heal Econ (United Kingdom).

[38] Parkinson F, Kent SJW, Aldous C, Oosthuizen G, Clarke D (2014). The hospital cost of road traffic accidents at a South African regional trauma centre: a micro-costing study. Injury.

[39] Freeman M, Savva N, Scholtes S (2016). Economies of scale and scope in hospitals.

[40] Ministry of Health Republic of Uganda. Human Resources for Health Audit Report 2017/18. Available from: http://library.health.go.ug/publications/human-resources-health/human-resources-health-audit-report-201718. Accessed 8th Feb 2020.

[41] Chamberlain S, Stolz U, Dreifuss B, Nelson SW, Hammerstedt H, Andinda J (2015). Mortality related to acute illness and injury in rural Uganda: task shifting to improve outcomes. PLoS One.

