# Supplementary material for: The costs of delivering emergency care at regional referral hospitals in Uganda: a micro-costing study
Source: BMC Health Serv Res. 2021 Mar 16;21:232. doi: 10.1186/s12913-021-06197-7 (PMC7961167; doi:10.1186/s12913-021-06197-7)
Supplement: Supplementary file 1 — Additional file 1: Supplementary Table 1. Treatment processes for five sentinel conditions. [file 12913_2021_6197_MOESM1_ESM.docx]

Supplementary Materials

**Supplementary Table 1: Treatment processes for five sentinel conditions**

| **Condition** | **Possible common treatments** | Diagnostics |
| --- | --- | --- |
| RTI | Antibiotics | X -ray |
|  | Analgesia | CT Scan |
|  | Chest Drain | Blood transfusion |
|  | Wound Closure | Blood test |
|  | Fracture reduction & splinting |  |
|  | Needle decompression |  |
|  | Oral or Nasal Pharyngeal Airway |  |
|  | Oxygen |  |
|  | IV Fluids |  |
|  | Transfusion/ blood given |  |
|  | Hemorrhage control |  |
|  | Intubation |  |
| PPH |  |  |
|  | Oral or Nasal Pharyngeal Airway | Blood transfusion |
|  | Oxygen | Blood test |
|  | IV Fluids | X -ray |
|  | Transfusion/ blood given |  |
|  | Hemorrhage control |  |
|  | Intubation |  |
| Asthma |  | X -ray |
|  | Oral or Nasal Pharyngeal Airway |  |
|  | Antibiotics |  |
|  | Nebulisation |  |
|  | Oxygen |  |
| Pneumonia |  |  |
|  | Antibiotics | Blood test |
|  | Chest drain | X -ray |
|  | Oral or Nasal Pharyngeal Airway |  |
|  | IV fluids |  |
|  | Oxygen |  |
| Paediatric diarrhea |  |  |
|  | Oral rehydration | Blood test |
|  | IV fluids |  |
|  |  |  |
